# Analysis of the chloroplast genome and phylogenetic evolution of three species of *Syringa*

**DOI:** 10.1007/s11033-022-08004-w

**Published:** 2022-11-12

**Authors:** Chengjun Yang, Kai Wang, Hang Zhang, Qingjie Guan, Jian Shen

**Affiliations:** 1 Key Laboratory of Saline-Alkali Vegetation Ecology Restoration, Northeast Forestry University, Harbin, 150040 China; 2 Northeast Asia Biodiversity Research Center, Northeast Forestry University, Harbin, 150040 China; 3 China-Ukraine Agriculture & Forestry Technology Development and Application International Cooperation Joint Lab, Jiamusi University, Jiamusi, 154007 China

**Keywords:** *Syringa*, Phylogeny, Chloroplast genome

## Abstract

**Background:**

By the time our study was completed, the chloroplast genomes of *Syringa oblata, S. pubescents* subsp*. Microphylla,* and *S. reticulate* subsp*. Amurensi*s had not been sequenced, and their genetic background was not clear.

**The research content:**

In this study, the chloroplast genomes of *Syringa oblata*, *S. pubescents* subsp*. Microphylla, S. reticulate* subsp*. Amurensis*, and five other species of Syringa were sequenced for a comparative genomics analysis, inverted repeat (IR) boundary analysis, collinearity analysis, codon preference analysis and a nucleotide variability analysis. Differences in the complete chloroplast genomes of 30 species of *Oleaceae* were compared with that of *S. oblata* as the reference species, and *Ginkgo biloba* was used as the out group to construct the phylogenetic tree.

**Results:**

The results showed that the chloroplast genomes of *S. oblata, S. pubescents* subsp*. Microphylla,* and *S. reticulate* subsp*. Amurensi*s were similar to those of other angiosperms and showed a typical four-segment structure, with full lengths of 155,569, 160,491, 155,419, and protein codes of 88, 95, and 87, respectively. Because the IR boundary of *S. pubescents* subsp*. Microphylla* was significantly expanded to the large single copy (LSC) region, resulting in complete replication of some genes in the IR region, the LSC region of *S. pubescents* subsp*. Microphylla* was significantly shorter than those of *S. oblate* and *S. reticulate* subsp*. Amurensis*. Similar to most higher plants, these three species have a preference for their codons ending with A/T.

**Conclusions:**

We consider the genus *Syringa* to be a synphyletic group. The nucleotide variability and phylogenetic analyses showed that *Syringa* differentiated before *Ligustrum* and *Ligustrum* developed from *Syringa*. We propose removing the existing section division and directly dividing *Syringa* into five series.

**Supplementary Information:**

The online version contains supplementary material available at 10.1007/s11033-022-08004-w.

## Background

*Syringa* plants occupy an important position in Chinese gardens. *Syringa oblata*, *Syringa pubescents* subsp*. Microphylla,* and *Syringa reticulate* subsp*. Amurensis* belong to *Syringa* of Oleaceae [1]. According to the relative length of corolla tubes and sepals, syringa is classified into *Sect. Syringa* and *Sect. Ligustrina*. Accord to the different leaf shape and inflorescence, *Sect. Syringa* be divided into *Ser. Pinnatifoliae Rehd, Ser. Pubescentes (Schneid.) Lingelsh, Ser. Syringa* and *Ser. Villosae (Schneid.) Rehd.* However, this classification is controversial, and whether *Sect. Syringa* should be an independent section or a parallel series with the *Ser. Syringa* is the main point of controversy.

Chloroplasts are the photosynthetic organelles of green plants, and have functions of synthesizing starch, fatty acids, pigments, and proteins [2]. Chloroplasts are generally considered the origin of endosymbiosis. Photosynthesizing prokaryotic cyanobacteria were absorbed into cells by primitive eukaryotes with phagocytic ability, and gradually transitioned from parasitism to symbiosis, and became a eukaryote organelle that allowed solar energy to be used autonomously, and the transformation from heterotrophic to autotrophic occurred. The chloroplast has a complete genetic system called the chloroplast genome. The chloroplast genome of gymnosperms is generally inherited from the paternal line [3], while the chloroplast genome of most angiosperms is inherited from the maternal line. About 20% of the genome may be inherited from both parents or from the paternal line [4–6].

The sequence and structure of the chloroplast genome are relatively conserved. The chloroplast genome of most plants has a double-stranded ring structure, including four regions, as two inverted repeat regions (IRa/IRb), one large single copy region (LSC) and one small single copy region (SSC) in four parts. Genome length is relatively fixed at 12–18 kb [7]. As a higher organelle of plants, the chloroplast genome has a stable structure that is highly conserved, with a large number of copies that can be easily extracted and purified. A series of characteristics determine the important position and significance of the chloroplast genome in plant phylogeny, species identification, and genetic transformation. The chloroplast genome can be used as important research material to study species phylogeny, which plays an important role in determining the genetic relationships among related species. Applying the chloroplast genome at the population level provides clues to study the timing of differentiation and intraspecific intensity. Analyses with nuclear genome data provide a more comprehensive understanding of plant pollen transmission, migration pathways, and species evolutionary dynamics.

In this study, we sequenced the complete chloroplast genomes of *S. oblata, S. pubescents* subsp*. Microphylla,* and *S. reticulate* subsp*. Amurensis* and conducted comparative genomics of eight species of Oleaceae. We analyzed and combined the complete chloroplast genomes, LSC regions, SSC regions, IR regions, and introns of 30 species of Oleaceae published at the NCBI to construct a phylogenetic tree, and explored the phylogenetic relationships among the three species and their phylogeny in Oleaceae.

## Methods

### Plant materials

The plant materials required for this study were collected from the campus of Northeast Forestry University (45°43′ 16.83″ N, 126°38′ 2.04″ E) in July 2020. The fresh leaves of the three plants were quickly frozen in liquid nitrogen after collection, and then stored in a refrigerator at − 40 ℃. DNA extraction was carried out, and the extracted DNA was sent to Nanjing Jisihuiyuan Company for subsequent sequencing. The sequencing was submitted to NCBI database. The serial numbers of *Syringa oblata, S. reticulate* subsp *Amurensis* and *S. pubescents* subsp *Microphylla* is MT872639.1 MT872640.1 MT872641.1. The complete chloroplast genome sequences of the other five syringa species were obtained by NCBI database.

### Genome assembly and annotation

This study adopted the whole genome shotgun strategy to construct libraries with different insert fragments [8] using second-generation sequencing technology (next-generation sequencing), based on the HiSeq sequencing platform, to construct the libraries using paired-end (PaIRed-end, PE) sequencing [9]. After DNA extraction, purification, library construction and sequencing. The off-machine data were saved in the PaIRed-end FASTQ format, and the off-machine data were further filtered to remove the reads with connectors and the low quality in the sequencing data to ensure the quality of the subsequent information analysis [10]. Spades 3.12 was used to de-assemble the sequencing data with the adapter sequence removed to obtain the final assembly sequence [11]. OGDRAW (https://chlorobox.mpimp-golm.mpg.de/OGDraw.html) was used to make a chloroplast genome map [12].

### Genome feature analysis and comparative genome analytical method

Repetitive sequences are important genetic markers and are closely related to the origin and evolution of a species. Repetitive sequences have been divided into scattered repetition and tandem repetition types. Scattered repetitive sequences are repetitive sequences that are scattered in the genome. The scattered repetitions were analyzed using Reputer software [13], there is a special form of tandem repetition, which is also called SSR. SSRs often have natural polymorphisms, and cpSSR is the key component of a chloroplast genome repeat sequence analysis. The simple repetitive sequences were identified using MISA tools (parameters: 1-10 2-5 3-4 4-3 5-3 6-3) [14]. Codon preference was analyzed using the EMBOSS [15] (The European Molecular Biology Open Software Suite) and CodonW online programs, and the loss of genetic diversity reflected in the chloroplast genome was enhanced and showed a lower level of genetic diversity. Nucleotide diversity (π) was used to evaluate the level of population genetic diversity, using DnaSP software [16] to determine the local collinearity between genomes through multiple sequence alignment, and to perform similarity and a reanalysis of row, inversion, and other phenomena. The Mauve tool was used for the collinearity analysis. RAxML software was used to construct the maximum likelihood tree for the chloroplast genome [17].

## Results

### Genetic analysis

**Fig. 1:**
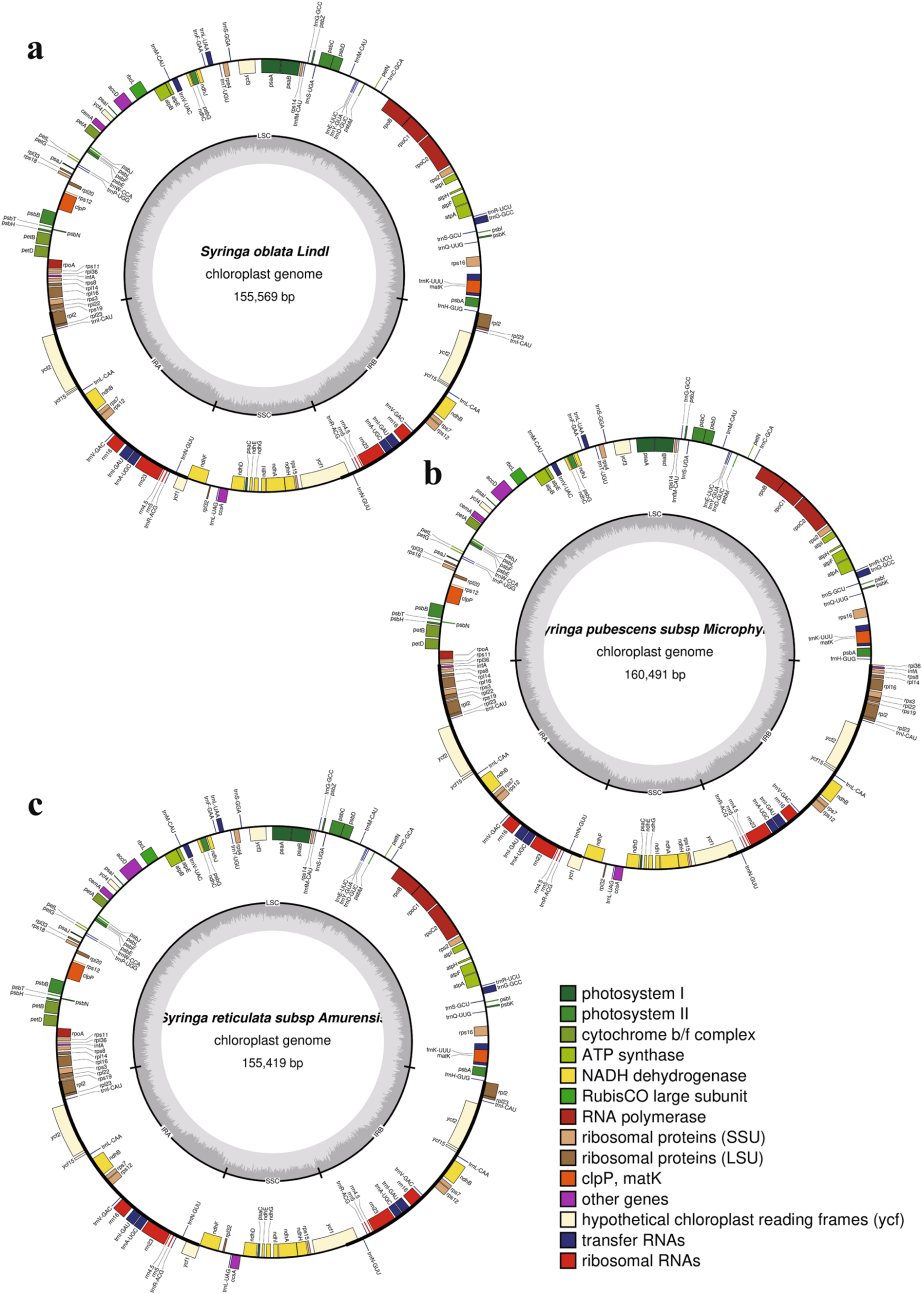
Gene map of the complete of three species of *Syringa*

Similar to other angiosperms, the chloroplast genome structures of the eight species of *Syringa* listed were all closed circular double-stranded DNA, each containing four partitions, one LSC region, and one SSC region with two IRs (Fig. 1). The length of seven genomes, except that of *S. pubescens* subsp*. Microphylla*, was about 15.5 kb, and GC content was 37.9%. The lengths of the LSC, SSC, and IR regions were 8.6, 1.7, and 2.5 kb, respectively, and GC contents were 36%, 32%, and 43% respectively. The total number of encoded genes was 130–132. *S. oblate* and *S. reticula*te subsp. *Amurensis* showed highly consistent chloroplast genome characteristics. The total length of the chloroplast genome of the two species was about 15.5 kb, the length of each partition was similar, and the numbers of tRNAs and rRNAs were 37 and 8, respectively. Only one protein-coding gene difference was detected in the chloroplast genomes of these two species (Table S1). The chloroplast genome of *S. oblata* contained 88 protein-coding genes, which had one more acetyl-CoA carboxylase gene (accD) than *S. reticulate* subsp*. Amurensis*. The chloroplast genome of *S. pubescens* subsp*. Microphylla* was significantly longer than that of the other seven species at 160,491 bp, as well as the length of the four partitions. The largest difference was found in the length of the LSC region of *S. pubescens* subsp*. Microphylla*, which was 82,890 bp, while the length of the LSC region of the other seven species was about 86 kb, which was significantly shorter than the other listed species. There was little difference in the length of the SSC regions of these species. The IR region of *S. pubescens* subsp*. Microphylla* was 29,958 bp, which was longer than that of the other seven species. The IR regions of the other seven species were 25–26 kb. The shortening of the LSC region and expansion of the IR region of *S. pubescens* subsp*. Microphylla* caused rpl14, rpl16, rpl22, rps19, rps3, rps8, infA, and other genes originally located in the LSC region to fall into the IR region. Thus, resulting in 95 protein-coding genes and 140 total genes. The gene types in the chloroplast genome of *S. pubescens* subsp*. Microphylla* were the same as those in *S. oblata*. Only quantitative differences in the three types of genes were detected, such as large ribosomal subunits, small ribosomal subunits, and translational initiation factors. The chloroplast genome of *S. oblata* contained only one copy of the rpl14, Rpl16, rpl22, rps19, rps3, rps8, and infA genes, whereas *S. pubescens* subsp*. Microphylla* contained two. These differences appeared between trnH-GUG and ycf2, and no other differences were detected in genes at other positions (Table S2).

### Expansion and contraction of the IR zone

**Fig. 2:**
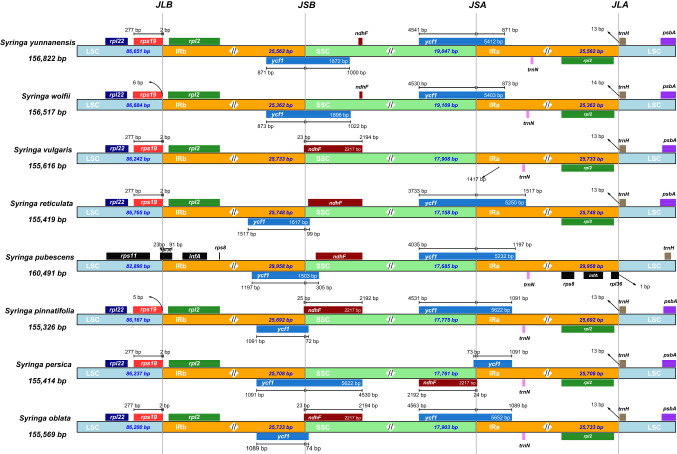
Expansion and contraction of the inverted repeat (IR) region in eight species of *Syringa*

Although the IR region of the chloroplast genome is the most conserved region, the contraction and expansion of its border region is a common phenomenon in the evolution of a chloroplast genome. This is also one of the most important factors for the length of the chloroplast genome. Among the eight species of *Syringa*, the borders of the LSC and IRb regions of seven species, except *S. pubescens* subsp*. Microphylla*, fell between the two genes, rps19 and rpl2. The rps11, rpl36, and infA genes were found near the boundary of the LSC and IRb regions in *S. pubescens* subsp. *Microphylla*, which was different from the other seven species. The rps19 gene of *S. oblata* and *S. reticulate* subsp*. Amurensis* was located on the boundary between the LSC and IRb regions, and both extended 2 bp into the IRb region. The ycf1 and ndhF genes were found near the borders of the SSC and IRb regions of *S. oblata*, *S. pubescens* subsp*. Microphylla*, and *S. reticulate* subsp*. Amurensis*. The lengths of the ycf1 gene extending into the SSC region were 74, 305, and 99 bp, respectively. The ndhF gene of *S. pubescens* subsp*. Microphylla* and *S. reticulate* subsp*. Amurensi*s fell completely in the SSC region, and the ndhF gene of *S. oblata* extended 23 bp into the IRb region, becoming a pseudogene. The rpl2 and trnH genes were found near the boundary between the LSC and IRa regions of the remaining seven species, except *S. pubescens* subsp*. Microphylla*, and they were close to the boundary between the LSC and IRa regions. The trnH gene of *Syringa wolfii* was 14 bp from the boundary between the LSC and Ira regions, and the remaining six species exhibited a distance of 13 bp. Three genes, such as rps8, infA, and rpl36, were detected at the boundary between the LSC and IRa regions of *S. pubescens* subsp*. Microphylla*. All three genes were located in the IRa region, and rpl36 was 1 bp from the boundary. The trnH gene was also found in the LSC region of *S. pubescens* subsp*. Microphylla* but it was distant from the boundary between the LSC and Ira regions (Fig. 2).

### Codon preference analysis

**Fig. 3:**
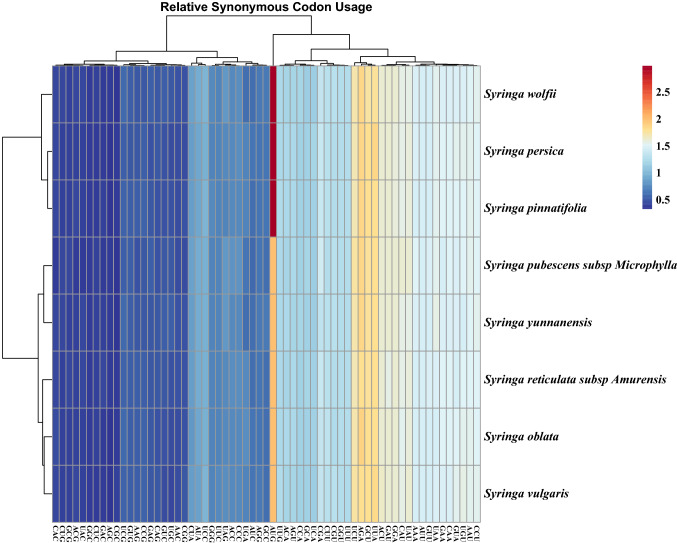
Heatmap of codon clustering for eight species of *Syringa*

26,620, 27,428, and 25,723 codons were found in the complete chloroplast genomes of *S. oblata, S. pubescents* subsp*. Microphylla,* and *S. reticulate* subsp*. Amurensis*, respectively. The most frequently used amino acid was Leu, and the smallest amino acid was Cys. All other amino acids have two or more codons, except Try, which has only one codon, and Arg, Leu and Ser have six codons. The codons of the eight species tended to end in A/U, which is the same as the codon preference of other angiosperm chloroplast genomes [18] (Fig. 3).

### Repeat sequence analysis

Repetitive sequences are important genetic markers and are closely related to the origin and evolution of a species. Interspersed and tandem repetitive sequences are the two main types of repetitive sequences. Interspersed repetitive sequences generally include four types, namely forward repeats, reverse repeats, palindrome sequences, and complement sequences. There are differences in the numbers and types of scattered repeating sequences in different species. The total number of interspersed repetitive sequences of *S. pubescents* subsp*. Microphylla* (n = 164) and *S. reticulate* subsp*. Amurensis* (n = 163) was far more than the other listed species, and only 46 were found in *S. oblata*. Among the eight species of *Syringa*, only complementary sequences were found in the chloroplast genomes of *S. microphylla* and *S. yunnanensis.* The chloroplast genome of *S. reticula*te subsp*. Amurensis* contained only forward (n = 135) and palindromic sequences (n = 29), excluding IRs and complementary sequences. All four scattered repeats (111, 32, 10, and 10) were found in *S. pubescents* subsp*. Microphylla*. No complementary sequences were detected in the chloroplast genome of *S. oblata*. Twenty forward repeats and 25 palindromic sequences were observed, which contained only one IR (Table S3).

**Fig. 4:**
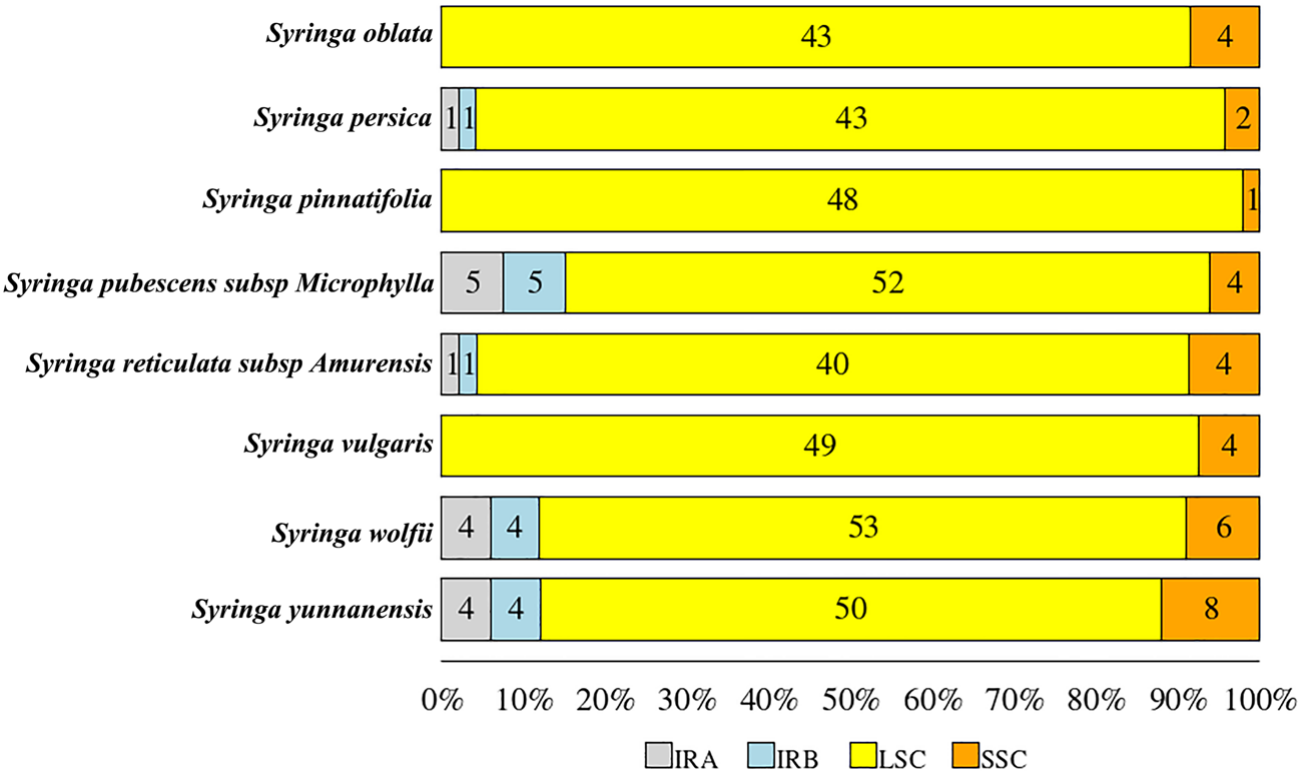
cpSSR distribution statistics of eight species of *Syringa*

A special form of tandem repeat is a simple tandem repeat, also called a simple sequence repeat (SSR). SSRs often have natural polymorphisms. cpSSR is the key content of a chloroplast genome repeat sequence analysis. The types and numbers of SSR sites in *S. oblata*, *S. pubescents* subsp*. Microphylla*, and *S. reticula*te subsp*. Amurensis* were studied. The results showed that the number of SSRs varied among the different species. Mononucleotide repeats account for the majority of SSRs, and these SSRs all appear in non-coding regions or protein-coding genes, and generally do not appear in tRNA or rRNA (Table S4). The numbers of mononucleotide SSRs in *S. oblata*, *S. pubescents* subsp*. Microphylla*, *S. reticulate* subsp*. Amurensis* were 35, 36 and 47, respectively. None of these three species contained pentanucleotide SSRs, and only *S. pubescents* subsp. *Microphylla* contained a hexanucleotides SSR. The SSRs of the three species were located in the protein coding or non-coding regions, and no SSR sites were found on tRNA or rRNA (Table S5). Among the eight species of *Syringa*, the SSRs were primarily located in the LSC region, with fewer distributed in the SSC and IR regions. Some species, such as *S. oblata*, *S. pinnatifolia*, and *S. vulgaris*, have no SSRs in the IR region. Totals of 43, 40, 52 SSRs were found in the LSC region, and four SSR sites were found in the SSC regions of *S. oblata*, *S. pubescents* subsp*. Microphylla*, and *S. reticulate* subsp*. Amurensis*. No SSR was found in the IR of *S. oblata*, and one and five SSRs were found in the IRs of *S. pubescents* subsp*. Microphylla* and *S. reticulate* subsp*. Amurensis*, respectively (Fig. 4). The complex nucleotide SSRs of the three species were all located in the non-coding region of the LSC region, and two trinucleotide ((TTA)4(ATA)4) SSRs of *S. reticulate* subsp*. Amurensis* only appeared in the IRa/IRb region. The trinucleotide SSRs of *S. oblate* and *S. pubescents* subsp*. Microphylla* occurred in the LSC region, and the four hexanucleotide SSRs of *S. pubescents* subsp*. Microphylla* all appeared in the IRa/IRb region. No hexanucleotides were detected in the chloroplast genomes of the other two species. SSRs mainly appear in several genes, such as matK, rps16, atpF, rpoC2, ycf3, atpB, rps12, ycf1, ycf2, and rpl16. No SSRs were found in the ycf1, ycf2, and rpl16 genomes of lilac. No SSRs were found in the ycf2 and rpl16 genes of *S. reticulate* subsp*. Amurensis.*

### Analysis of variations in the chloroplast genome

Many variations are found in chloroplast genome sequences. The variations in the genome are mainly divided into three categories: 1. single nucleotide variations, (usually called single nucleotide polymorphisms, that is, the difference in a single DNA base, referred to as a SNP); SNPs only involve the variation of a single base, which can be caused by transition or transversion of a single base, or by the insertion or deletion of bases; 2. small indels (insertion and deletion); that is, insertion or deletion of a small sequence < 50 bp at a certain position in the genome; 3. large structural variations (SVs), including those with lengths ≥ 50 bp, insertion or deletion of long fragments, shift inversion, copy number variations, and some more complicated forms of variation. The second and third types of variation are also called SVs to distinguish them from SNP variation. The analytical results are shown in Table S6 using *S. oblata* as the reference species. The maximum number of SNPs in *S. reticulate* subsp*. Amurensis* was 1,642, with 311 indels. In total, 372 indels were present in *S. pubescens* subsp*. Microphylla*, with 1588 SNPs, indicating that the two are quite different from the chloroplast genome of *S. oblata*, and their genetic relationship is relatively distant; the numbers of SNPs and indels in *S. vulgaris* were 171 and 49 respectively. In traditional taxonomy, *S. reticula*te subsp*. Amurensis* belongs to Sect. *Ligustrina,* whereas the other listed species belong to *Sect. Syringa. S. vulgaris* and *S. oblata* belong to *Ser. Syringa, and S. pubescens* subsp*. Microphylla* belongs to *Ser. Pubescentes (Schneid.) Lingelsh.* The results of variations in the chloroplast genome verified this traditional classification result (Table S6).

### Collinearity analysis

**Fig. 5:**
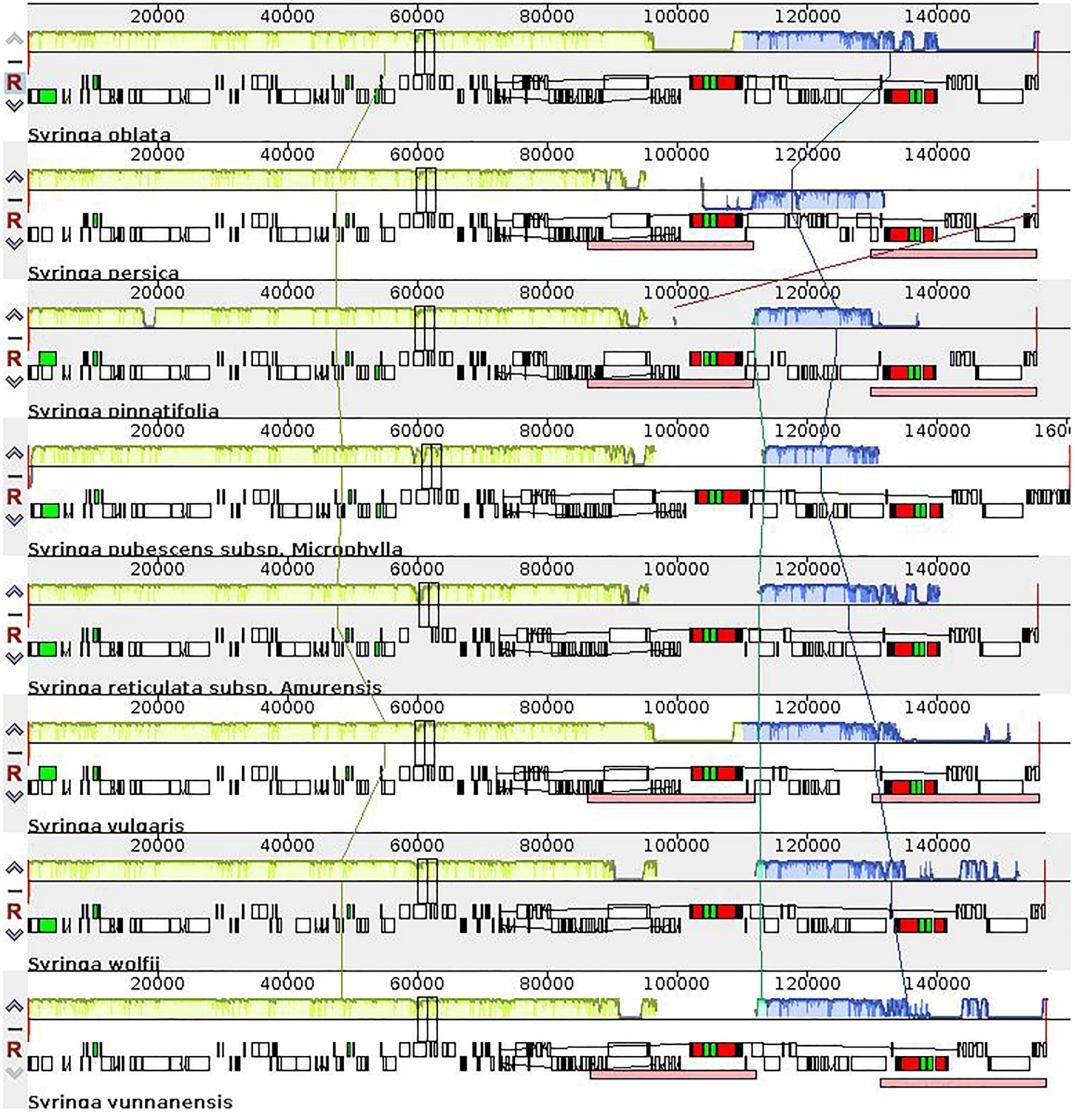
Collinear analysis of eight species of *syringa*

A collinearity analysis is performed to determine the local collinearity blocks between genomes through multiple sequence comparisons, and analyze the similarities, rearrangements, and inversions of the collinearity blocks to illustrate the events that occur during species evolution. Blocks of the same color connected by the same line represent similar gene segments of different species. Organisms increase the length of gene segments to follow changes in the evolutionary process. The collinearity analysis of eight species of *Syringa* is shown in the Fig. 4, considering *S. oblata* as the benchmark species. Only one gene segment of *S. persica* was rearranged, and the gene segments of the other seven *Syringa* species increased or decreased significantly compared with those of *S. oblata* (Fig. 5).

### Chloroplast variability analysis

**Fig. 6:**
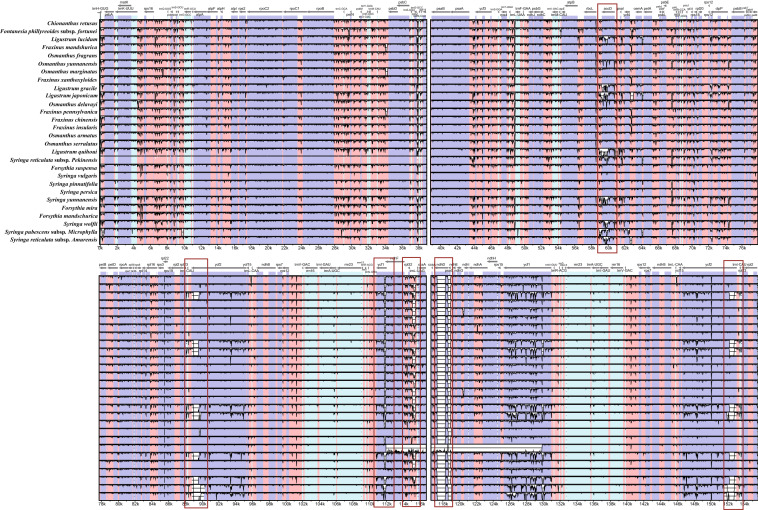
Comparative analysis of the chloroplast genomes of 29 species of Oleaceae

Using *S. oblata* as the benchmark species, the Vista tool was used to conduct a genome-wide comparative analysis of 28 Oleaceae species. The results showed that the variation in the non-coding region was greater than that in the coding region, the SC region was larger than the IR region, and the RNA part of the gene was the most conserved. The genes were divided into three categories based on variations in the different genes from different species. The first parts of the genes were quite different among some species. For example, the *accD*, *ycf1*, and *ycf2* genes of some species were quite different from those of lilac. The second parts of the genes were also quite different in all of the listed species, such as the *ndhD* and *psaC* genes. The third part was the more conserved genes, such as *pasA*, *pasB*, and other genes, which differed highly among the listed species. The first and second types of genes can be used for the development of genetic barcodes (Fig. 6).

### Phylogenetic analysis

**Fig. 7:**
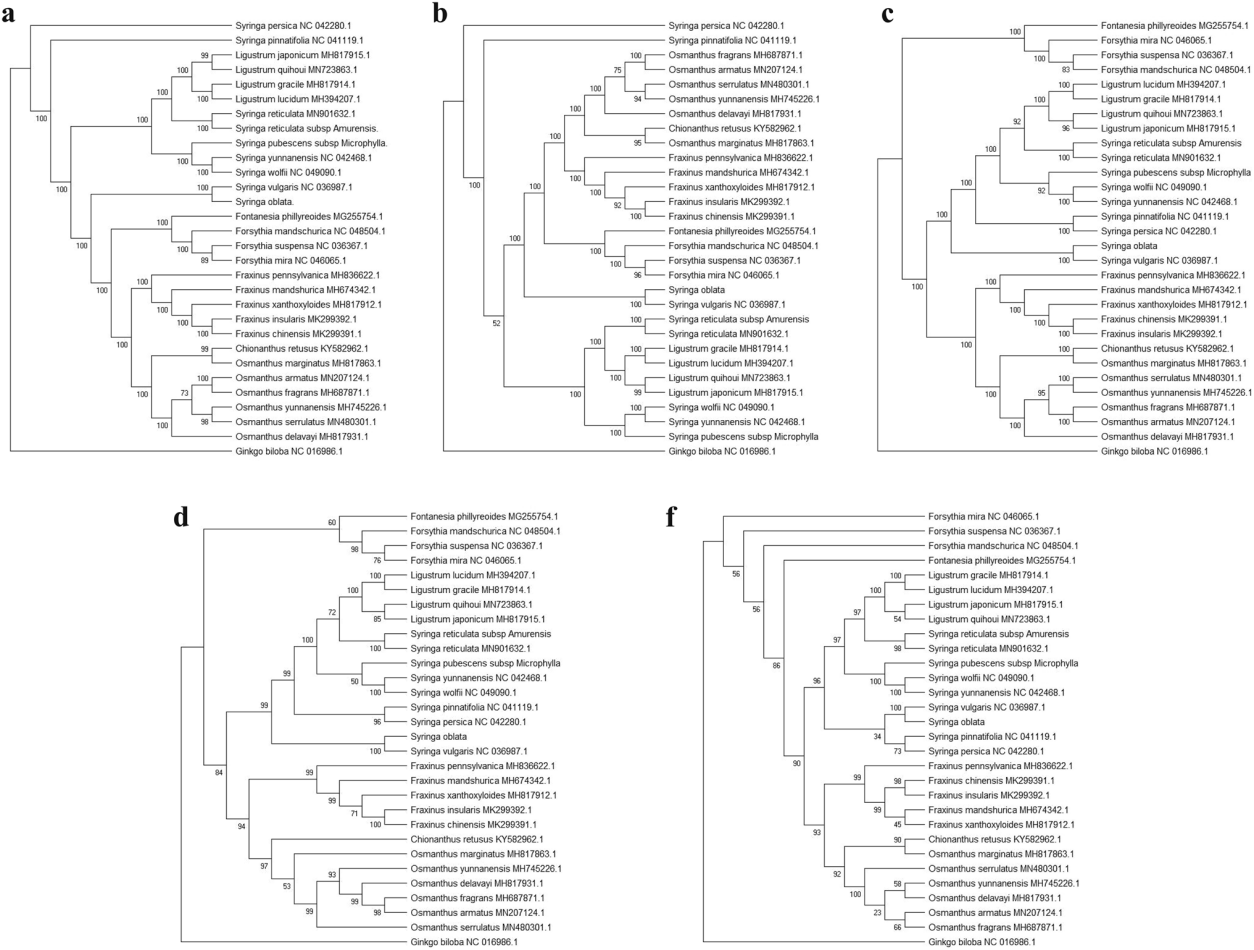
A phylogenetic tree with Ginkgo biloba as the out group was constructed based on five parts of the chloroplast genome of 29 species of Oleaceae

We use *Ginkgo biloba* as the out group and constructed a phylogenetic tree with the chloroplast genomes, CDS regions, introns, LSC regions, and IR region of *S. oblata, S. pubescents* subsp*. Microphylla, S. reticulate* subsp*. Amurensis* and 26 other species of Oleaceae published at the NCBI. The five phylogenetic trees all showed that *Syringa* differentiated before *Ligustrum*, indicating that *Ligustrum* was derived from *Syringa* (Fig. 7).

The phylogenetic tree of the CDS region was similar to the phylogenetic tree of the complete chloroplast genome, as both show that *S. persica* and *S. pinnatifolia* were sequentially separated from the whole of Oleaceae, indicating that the two are evolutionarily primitive. *Ligustrum* and some *Syringa* aggregated into one large branch, and the lower branches belonged to *Ligustrum* and *Ser. Villosae (Schneid.) Rehd* of *Sect. Syringa*, including *Syringa wolfii*. *Syringa yunnanensis* and *Ser. Pubescentes (Schneid.) Lingelsh*, juxtaposed with *S. reticulata* subsp. *Amurensis*, which belonged to *Sect. Ligustrina Rupr*. In other branches, *Ser. Syringa* represented by *S. vulgari*s and *S. oblata* was on the same branch with *Fraxinus*, *Forsythia*, and *Osmanthus*, and the relationship was distant from *Ser. Pubescentes (Schneid.) Lingelsh* represented by *S. pubescens* subsp*. Microphylla,* although the two both belonged to *Sect. Syringa* in the existing classification of *Syringa*.

The results of the evolutionary tree of introns and the LSC regions were similar. *Forsythia* was the first to differentiate. *Syringa* and *Ligustrum* aggregated on one branch, and several other genera aggregated on a large branch. Among the *Syringa* and *Ligustrum* branches*, Syringa* differentiated earlier than *Ligustrum*. The sections and series in *Syringa* were aggregated separately. *Sect. Syringa* was divided into groups before *Sect. Ligustrina*. The differentiation order of *Sect. Syringa* is *Ser. Syringa, Ser. Pinnatifoliae Rehd,* and *Ser. Villosae (Schneid.) Rehd. Ser. Pubescentes (Schneid.) Lingelsh.* In the IR zone of the evolutionary tree, *Forsythia* first differentiated and *Syringa* and *Ligustrum* converged into one branch. *Ser. Pinnatifoliae Rehd* and *Ser. Syringa* converged into one branch juxtaposed with other lines of *Syringa* and *Ligustrum*. In the lower branch, *Ligustrum* and *Sect. Ligustrina* were juxtaposed with *Ser. Villosae (Schneid.) Rehd* and *Ser. Pubescentes (Schneid.) Lingelsh* on one branch.

The five-part phylogenetic tree shows that *Ligustrum* has the closest relationship with *Syringa* in Oleaceae, and it maybe evolved from *Syringa*, which is older than *Ligustrum*. The existing systematic classification system within *Syringa* has certain problems, and the results support converting the genus *Syringa* into five series.

## Discussion

The chloroplast genomes of different species differed in length and the number of encoded proteins, but the range of variation was rather small. The chloroplast genome of most angiosperms is relatively fixed at 12–17 kb in length and is divided into four partitions. The IR and SSC regions are relatively conserved, and the LSC region is the most variable. The number of genes encoding proteins was relatively fixed at about 85; four rRNAs were present, and GC% content was relatively fixed at about 38%. Studies have shown that some species of Amaryllidaceae [19] and Rosaceae [20] have similar genetic characteristics, such as the length of the chloroplast genome and the number of encoded proteins. The IR region is the most conserved region in the plant chloroplast genome. The length, structure, and boundary between the IR and SC regions are all highly conserved. Expansion and contraction of the IR region are important factors that change the length of the plant chloroplast genome, and they are generally divided into two types. Expansion and contraction of the IR region of most species are reflected in the small deviation in the IR/SC boundary within a few fixed genes. This deviation may cause some genes to become pseudogenes. *Labiata*e [21] and *Vitis* [22, 23] have φycf1 and φrps19 pseudogenes in the IR/SC boundary, and the IR/SSC boundary was more conserved than the IR/LSC boundary, indicating that the main direction of expansion of the IR region is towards the LSC area. The LSC/IRb boundary generally fell on or near rps19, rpl2, or rpl22. IRb/SSC generally fell on ycf1 or between ycf1 and ndhF, SSC/IRa generally fell on ycf1, and the IRa/LSC boundary generally fell on or near rps19, rpl2, rpl12, and trnH. The IRb/SSC boundary of *Ricinu*s [24], *Gossypium *[25], *Gossypium* [22] and other species falls on or near the ycf1 gene, and the SSC/IRa boundary falls on the ycf1 gene. The IR/LSC boundaries of the chloroplast genomes of the above are different. The lcs/IRb boundary of *Ricinus* falls on rpl22, and the IRa/LSC boundary falls on or near rpl22 and trnH. The *Gossypium* and *Viti*s lcs/IRb boundary falls on rps19, and the IRa/LSC boundary falls between rps19 and trnH. In this case, expansion of the IR region usually only changes from a few to dozens of base pairs, and does not cause a change in the number of genes. The other is the IR boundary crossing to other genes. For example, expansion and contraction of the IR region of *S. oblate* and *S. reticulate* subsp*. Amurensis* allow the IR boundary to change tens or even a few bps on a few genes, while this region in *S. pubescents* subsp*. Microphylla* expanded to the LSC region into the rpl36 gene. Several genes originally located in the LSC region were included in the IR region, resulting in complete replication of the gene, and the total number of genes in the chloroplast genome changed accordingly.

cpSSR molecular markers were first developed by Powell in 1995 as a phylogenetic hotspot [26]. The density of the cpSSRs contained in different regions of the chloroplast genome is different because of the uneven distribution of molecular markers among different taxa. Other characteristics have become excellent material for phylogenetic research. SSRs contain multiple types of repetitive sequences, including single nucleotide, dinucleotide, trinucleotide, tetranucleotide, pentanucleotide, hexanucleotide, and complex nucleotide repetitive sequences. The results of published plant chloroplast genome analyses show that cpSSRs with a single nucleotide repeat sequence of more than eight bases are more common and more numerous in the non-coding regions of most plants. Jakobsson and Ebert reported that CpSSRs with a single-base repeat sequence of fewer than seven bases cannot be used to distinguish between species [27, 28]. The number of cpSSRs contained in different groups of species was also quite different. Studies have shown that some species of Nymphaeaceae and *Cuscuta* contain fewer cpSSRs, while some species of Cruciferae contain more cpSSRs [29].

Traditional systematic classification is based on the morphological characteristics of *Syringa*. Rehder is divided into two subgenera under *Syringa* (*Sect. Syringa and Sect. Ligustrina*) [30]. The current classification divides the genus *Syringa* into two subgenera, namely *Sect. Syringa and Sect. Ligustrina*, among which *Sect. Syringa* consists of *Ser. Pinnatifoliae Rehd, Ser. Pubescentes (Schneid.) Lingelsh, Ser. Syringa,* and *Ser. Villosae (Schneid.) Rehd*. Chen Jinyong [31] reported shape and multivariate analyses that the genus *Syringa* is divided into two section s, two series, 12 species, and 13 subspecies, among which the *Sect. Ligustrina* contains one species, three subspecies, and *Sect. Syringa* contains a terminal inflorescence system and a lateral inflorescence system and includes five species and five subspecies and six species and 5 subspecies, respectively. He Miao and Zhuo Lihuan selected 11 species of *Syringa* from northeastern China. After a phylogenetic analysis based on 25 morphological characteristics, *Sect. Ligustrina* belonged to *Sect. Syringa,* the division of the section was removed, and *Syringa* was directly divided into five series, namely *Ser. Pinnatifoliae Rehd. Ser. Pubescentes (Schneid.) Lingelsh. Ser. Syringa, Ser. Villosae (Schneid.) Rehd*, *and Ser. Ligustrina Rupr.* [32]. Gao Yan et al. studied the relationship between the leaf epidermis characteristics of seven wild species of *Syringa* and the environmental adaptation mechanism and systematics, and inferred that the relationships among *Ser. Pinnatifoliae Rehd. Ser. Pubescentes (Schneid.) Lingelsh. Ser. Villosae (Schneid.) Rehd.* are closer [33]. The results of Kim’s restriction fragment length polymorphism technology using cpDNA and Qin Xiangkun’s isozyme technology support division of the genus into two sections [34, 35]; Gao Hongxiao et al. used amplified fragment length polymorphism analysis to remove the division of the sections, and reported that *Syringa* should be directly divided into four series [36]. Li et al. reached a similar conclusion by studying the ITS and ETS of rDNA [37]. In the present study, the phylogenetic results were similar to the result of He Miao and Zhuo Li huan, and directly support dividing *Syringa* into five series.

## Conclusion

In this study, the complete chloroplast genome sequences and annotations of *S. oblata, S. pubescents* subsp*. Microphylla,* and *S. reticulate* subsp*. Amurensis* were described and compared with five other species of *Syringa*. A comparative genomics analysis, IR region boundary analysis, collinearity analysis, and codon preference analysis were performed. Using *S. oblata* as the reference species, 30 species of Oleaceae were used as materials to analyze variability in the entire chloroplast genome. A phylogenetic tree was constructed using *G. biloba* as the out group, as well as the CDS regions, IR regions, LSC regions, and introns. The results support the existing classification of Oleaceae, sort out the phylogenetic relationships among the genera in Oleaceae, and sort out the group divisions within *Syringa*.

## Supplementary Information

Below is the link to the electronic supplementary material.

Supplementary file1 (DOCX 21 kb)

## Data Availability

The authors declare that we have submitted the sequence information to NCBI with the numbers MT872639.1 MT872640.1 MT872641.1. We have released the above data.

## Abbreviations

LSC: Large single copy regions
SSC: Small single copy regions
IR: Inverted repeat regions
CDS: Coding sequence
SSR: Simple sequence repeat
cpSSR: Chloroplast simple sequence repeat
mono: Mononucleotide repeats
di: Dinucleotide repeats
tri: Trinucleotide repeats
tetra: Tetranucleotide repeats
penta: Pentanucleotide repeats
hexa: Hexanucleotides repeats
complex: Complexnucleotide repeats
SNP: Single nucleotide polymorphism;
InDel: Insertion or deletion of small DNA sequences < 50 bp
Ts: Number of SNPS converted;
Tv: Number of switched SNPS
