# Supplementary material for: Analysis of the chloroplast genome and phylogenetic evolution of three species of Syringa
Source: Mol Biol Rep. 2022 Nov 12;50(1):665–77. doi: 10.1007/s11033-022-08004-w (PMC9884260; doi:10.1007/s11033-022-08004-w)
Supplement: Supplementary file 1 — Supplementary file1 (DOCX 21 kb) [file 11033_2022_8004_MOESM1_ESM.docx]

Table S1: Chloroplast genome information of eight species of *Syringa*

| Species | *Syringa oblata* | *Syringa persica* | *Syringa pinnatifolia* | *Syringa pubeSCens_*  Subsp *Microphylla* | *Syringa reticulata_*  Subsp *Amurensis* | *Syringa*  *vulgaris* | *Syringa*  *wolfii* | *Syringa yunnanensis* |
| --- | --- | --- | --- | --- | --- | --- | --- | --- |
| Size | 155569 | 155414 | 155326 | 160491 | 155419 | 155616 | 156517 | 156822 |
| PCGs | 88 | 88 | 86 | 95 | 87 | 86 | 87 | 88 |
| tRNAs | 37 | 35 | 37 | 37 | 37 | 36 | 36 | 35 |
| rRNAs | 8 | 8 | 8 | 8 | 8 | 8 | 8 | 8 |
| Genes | 133 | 131 | 131 | 140 | 132 | 130 | 131 | 131 |
| GC% | 37.92% | 37.97% | 37.93% | 37.89% | 38.22% | 37.89% | 37.95% | 37.95% |
| Length(LSC) | 86200 | 86237 | 86167 | 82890 | 86765 | 86242 | 86684 | 86651 |
| Length(SSC) | 17903 | 17761 | 17775 | 17685 | 17158 | 17908 | 19109 | 19047 |
| Length(IR) | 25733 | 25708 | 25692 | 29958 | 25748 | 25733 | 25362 | 25562 |
| GC%(LSC) | 35.96 | 36.03 | 35.98 | 36.16 | 36.33 | 35.93 | 36.13 | 36.14 |
| GC%(SSC) | 32.12 | 32.17 | 32.08 | 32.52 | 33.03 | 32.1 | 32.57 | 32.6 |
| GC%(IR) | 43.21 | 43.23 | 43.24 | 41.87 | 43.15 | 43.21 | 43.1 | 43.02 |

Table S2: Contents of the chloroplast genome of *Syringa oblata*

| Category | Group | Genes |
| --- | --- | --- |
| Photosynthetic |  |  |
|  | Subunits of photosystem I | psaA,psaB,psaC,psaI,psaJ |
|  | Subunits of photosystem II | psbA,psbB,psbC,psbD,psbE,psbF,psbG,  psbH,psbI,psbJ,psbK,psbL,psbM,psbN,psbT,psbZ |
|  | Subunits of NADH dehydrogenase | ndhA,ndhB(x2),ndhC,ndhD,ndhE,ndhF,ndhG,ndhH,ndhI,ndhJ |
|  | Subunits of cytochrome b/f complex | petA,petB,petD,petG,petL,petN |
|  | Subunits of ATP synthase | atpA,atpB,atpE,atpF,atpH,atpI |
|  | large subunit of RubiSCO | rbcL |
| Self-replication |  |  |
|  | Large subunit of ribosomal | rpl14*,rpl16*,rpl2(x2),rpl20,rpl22*,  rpl23(x2),rpl32,rpl33,rpl36 |
|  | Samll subunit of ribosomal | rps11,rps12(x2),rps14,rps15,rps16,  rps18,rps19*,rps2,rps3*,rps4,rps7(x2),rps8* |
|  | Subunits of RNA polymerase | rpoA,rpoB,rpoC1,rpoC2 |
|  | Ribosomal RNAs | rrn16(x2),rrn23(x2),rrn4.5(x2),rrn5(x2) |
|  | Transfer RNAs | trnA-UGC(x2),trnC-GCA,trnD-GUC,trnE-UUC,trnF-GAA,  trnG-GCC(x2),trnH-GUG,trnI-CAU(x2),trnI-GAU(x2),trnK-UUU,  trnL-CAA(x2),trnL-UAA,trnL-UAG,trnM-CAU(x2),trnN-GUU(x2),trnP-UGG,  trnQ-UUG,trnR-ACG(x2),trnR-UCU,trnS-GCU,trnS-GGA,trnS-UGA,trnT-UGU,  trnV-GAC(x2),trnV-UAC,trnW-CCA,trnY-GUA,trnfM-CAU |
|  | Tanskational initiation factor | infA* |
| Other |  |  |
|  | Protease | clpP |
|  | Maturase | matK |
|  | Envelope membrance protein | cemA |
|  | c-type cytochrome synthesis gene | ccsA |
|  | Subunit of Acetyl-CoA-carboxylase | accD* |
|  | Hypothetical chloroplast | ycf1(x2),ycf15(x2),ycf2(x2),ycf3,ycf4 |
|  | reading frames | psaA,psaB,psaC,psaI,psaJ |

Table S3: Interspersed repetitive sequences types of eight species of *Syringa*

| type | forward | palindromic | reverse | complement | Total |
| --- | --- | --- | --- | --- | --- |
| *Syringa oblata* | 20 | 25 | 1 | 0 | 46 |
| *Syringa persica* | 20 | 27 | 1 | 0 | 48 |
| *Syringa pinnatifolia* | 20 | 25 | 0 | 0 | 45 |
| *Syringa pubescents* subsp *Microphylla* | 111 | 32 | 10 | 10 | 163 |
| *Syringa reticulata* subsp *Amurensis* | 135 | 29 | 0 | 0 | 164 |
| *Syringa vulgaris* | 20 | 26 | 1 | 0 | 47 |
| *Syringa wolfii* | 50 | 35 | 3 | 0 | 88 |
| *Syringa yunnanensis* | 59 | 46 | 3 | 1 | 109 |

Table S4: cpSSR distribution statistics of eight species of *Syringa*

| type | PCG | tRNA | rRNA | noncoding |
| --- | --- | --- | --- | --- |
| *Syringa oblata* | 20 | 0 | 0 | 27 |
| *Syringa persica* | 17 | 0 | 0 | 30 |
| *Syringa pinnatifolia* | 14 | 0 | 0 | 35 |
| *Syringa pubescents* subsp *Microphylla* | 32 | 0 | 0 | 34 |
| *Syringa reticulate* subsp *Amurensis* | 24 | 0 | 0 | 22 |
| *Syringa vulgaris* | 19 | 1 | 0 | 33 |
| *Syringa wolfii* | 35 | 0 | 0 | 32 |
| *Syringa yunnanensis* | 36 | 0 | 0 | 30 |

Table S5: cpSSR type statistics of eight species of *Syringa*

| Type | mono- | di- | tri- | tetra- | penta- | hexa- | complex |
| --- | --- | --- | --- | --- | --- | --- | --- |
| *Syringa oblata* | 35 | 3 | 1 | 6 | 0 | 0 | 2 |
| *Syringa persica* | 38 | 3 | 1 | 4 | 0 | 0 | 1 |
| *Syringa pinnatifolia* | 38 | 4 | 1 | 5 | 0 | 0 | 1 |
| *Syringa pubescens* subsp *Microphylla* | 47 | 3 | 7 | 3 | 0 | 4 | 2 |
| *Syringa reticulata* subsp *Amurensis* | 36 | 3 | 2 | 4 | 0 | 0 | 1 |
| *Syringa vulgaris* | 38 | 2 | 3 | 6 | 1 | 0 | 3 |
| *Syringa wolfii* | 43 | 4 | 6 | 5 | 3 | 2 | 4 |
| *Syringa yunnanensis* | 46 | 4 | 4 | 4 | 2 | 2 | 4 |

Table S6: Analysis of chloroplast variability in eight species of *Syringa*

| ID | SNP | INDEL | Ts | Tv | INS | DEL |
| --- | --- | --- | --- | --- | --- | --- |
| *Syringa persica* | 909 | 272 | 411 | 498 | 98 | 174 |
| *Syringa pinnatifolia* | 881 | 219 | 366 | 515 | 106 | 113 |
| *Syringa pubeSCens* subsp *Microphylla* | 1588 | 372 | 598 | 990 | 222 | 150 |
| *Syringa reticulata* subsp *Amurensis* | 1642 | 311 | 608 | 1034 | 158 | 153 |
| *Syringa vulgaris* | 171 | 49 | 62 | 109 | 33 | 16 |
| *Syringa wolfii* | 1333 | 303 | 543 | 790 | 168 | 135 |
| *Syringa yunnanensis* | 1367 | 310 | 558 | 809 | 171 | 139 |

Note: SNP: single nucleotide polymorphism, only involving the replacement of a single nucleotide; InDel: insertion or deletion of small DNA sequences < 50 bp; Ts: Number of SNPS converted; Tv: Number of switched SNPS.
